# Rehabilitation for life: the effect on physical function of rehabilitation and care in older adults after hip fracture—study protocol for a cluster-randomised stepped-wedge trial

**DOI:** 10.1186/s13063-022-06321-w

**Published:** 2022-05-07

**Authors:** Jonas Ammundsen Ipsen, Lars T. Pedersen, Bjarke Viberg, Birgitte Nørgaard, Charlotte Suetta, Inge H. Bruun

**Affiliations:** 1 Department of Physical Therapy and Occupational Therapy, Lillebaelt Hospital, University Hospital of Southern Denmark, Odense, Denmark; 2 Department of Regional Health Research, University of Southern Denmark, Odense, Denmark; 3 Department of Health Education, University College South Denmark, Odense, Denmark; 4 Department of Orthopaedic Surgery and Traumatology, Lillebaelt Hospital, University Hospital of Southern Denmark, Odense, Denmark; 5 Department of Public Health, University of Southern Denmark, Odense, Denmark; 6 Department of Geriatric and Palliative Medicine, Bispebjerg and Frederiksberg Hospitals, University of Copenhagen, Copenhagen, Denmark; 7 Department of Medicine, Herlev and Gentofte Hospitals, University of Copenhagen, Copenhagen, Denmark

**Keywords:** Hip fracture, Rehabilitation, Care, Between sectors, Empowerment, Physical function, Stepped-wedge cluster randomised controlled trial

## Abstract

**Background:**

A hip fracture is a serious event for older adults, given that approximately 50% do not regain their habitual level of physical function, and the mortality rate is high, as is the number of readmissions. The gap in healthcare delivery, as separated into two financial and self-governing sectors, might be a contributing cause of inferior rehabilitation and care for these patients. Therefore, we aim to assess the effect of continuous and progressive rehabilitation and care across sectors for older adults after hip fracture.

**Methods/design:**

The project is designed as a stepped-wedge cluster randomised controlled trial. The study population of patients are older adults 65 years of age and above discharged after a hip fracture and healthcare professionals in primary and secondary care (municipalities and hospitals). Healthcare professionals from different sectors (hospital and municipalities) will be engaged in the empowerment-orientated praxis, through a workshop for healthcare professionals with knowledge sharing to the older adults using a digital health application (app). The rehabilitation intervention consists of 12 weeks of progressive resistance exercises initiated 1–2 days after discharge. To improve communication across sectors, a videoconference involving the patient and physiotherapists from both sectors will be conducted. On day, 3 after discharge, an outreach nurse performs a thorough assessment including measurement of vital signs. A hotline to the hospital for medical advice is a part of the intervention. The intervention is delivered as an add-on to the usual rehabilitation and care, and it involves one regional hospital and the municipalities within the catchment area of the hospital. The primary outcome is a Timed Up and Go Test 8 weeks post-surgery.

**Discussion:**

Using a stepped-wedge design, the intervention will be assessed as well as implemented in hospital and municipalities, hopefully for the benefit of older adults after hip fracture. Furthermore, the collaboration between the sectors is expected to improve.

**Trial registration:**

The study is approved by the Regional Scientific Ethics Committees of Southern Denmark (S-20200070) and the Danish Data Protection Agency (20-21854). Registered 9 of June 2020 at ClinicalTrials.gov, NCT04424186.

**Supplementary Information:**

The online version contains supplementary material available at 10.1186/s13063-022-06321-w.

## Administrative information

Please see Table 1.

**Table 1:** Administrative information

Title	Rehabilitation for life: the effect on physical function of rehabilitation and care in older adults after hip fracture—study protocol for a cluster-randomised stepped-wedge trial
Trial registration	ClinicalTrials.gov Identifier: NCT04424186
Protocol version	Protocol version number 1 date 10.11.2020
Funding	The project is funded by the National Association of Municipalities, the Region of Southern Denmark, the Novo Nordisk Foundation, the Association of Danish Physiotherapists, and the Research Council of Lillebaelt Hospital - University Hospital of Southern Denmark, Denmark.
Author details	1. Department of Physical Therapy and Occupational Therapy, Lillebaelt Hospital, University Hospital of Southern Denmark 2. Department of Regional Health Research, University of Southern Denmark 3. Department of Health Education, University College South Denmark 4. Department of Orthopaedic Surgery and Traumatology, Lillebaelt Hospital, University Hospital of Southern Denmark 5. Department of Public Health, University of Southern Denmark, Denmark 6. Department of Geriatric and Palliative Medicine, Bispebjerg and Frederiksberg Hospitals, University of Copenhagen, Denmark 7. Department of Medicine, Herlev and Gentofte Hospitals, University of Copenhagen, Denmark
Name and contact information for the trial sponsor	Kolding Hospital a part of Lillebaelt Hospital - University Hospital of Southern Denmark. Main phone number: +45 76 36 20 00
Role of sponsor	The contents of the published materials are solely the responsibility of the sponsor, Lillebaelt Hospital, and the individual authors identified and do not reflect the views of funders. Neither funders nor sponsor will have a role in the study design, data collection, data analysis, data interpretation, or writing of the reports. The trial will be completed indecently by the administering organisation and funders.

## Background and rationale

A hip fracture is a serious event for older adults since approximately 50% do not regain their habitual level of physical function thus, acquiring new or additional need for care [1, 2]. Furthermore, when compared to an age-matched group, the 1-year mortality increases threefold and the quality of life is reduced [2, 3]. The 30-day readmission rate after a hip fracture is as high as 16–19% [4, 5].

For older adults, it is well-known that poor mobilisation and reduced activity during and after hospitalisation trigger loss of muscle mass that moreover is associated to increased mortality [3]. To reduce mortality, early detection of illness and sufficient pain management has been identified as important **[**6**,** 7**]**. Insufficient pain management is associated to an increased risk of complications, morbidity, and mortality and also impedes physical activity [8]. Nevertheless, continuous and progressive rehabilitation, as well as the detection of critical illness and complications, is lacking across the sectors in a healthcare system divided into two financial and self-governing sectors.

In Denmark, the average length of stay is 5–7 days for hip fracture patients [7]. Rehabilitation in the primary sector must be initiated within 7 days after discharge. However, usual care does not include systematic assessment including vital signs measurement. Furthermore, various exercise regimes are used depending on the sectors, and the regimes are usually not specified in terms of intensity or progression. Communication and cooperation between sectors are also lacking, although the older adults express a need for increased involvement [9].

To impede functional decline and lower mortality and readmission rates, continuous and progressive rehabilitation and care across sectors are needed. This study introduces an empowerment-orientated praxis focusing on continuous rehabilitation and care, as well as optimised communication and cooperation between sectors.

### Objective

This study aims to assess the effect of continuous and progressive rehabilitation and care across sectors for older adults following a hip fracture.

### Trial design

**Fig. 1:**
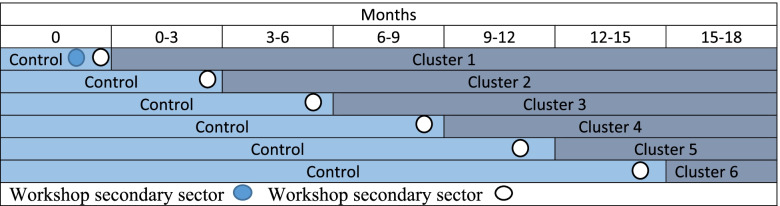
Overview of the clusters and the crossover from control to intervention

The protocol describes a cluster randomised stepped-wedge trial. It has a superiority design, a 1:1 allocation ratio, and the time interval for each step is set to three months, as illustrated in Fig. 1. The study protocol follows the Standard Protocol Items: Recommendations for Interventional Trial (SPIRIT) checklist (see Additional file 1) [10, 11]. A trial registration dataset is reported in Table 1.

## Methods

### Study setting

The trial will involve a regional hospital in Denmark and all six municipalities in the hospital’s catchment area. The municipalities serve a mixed urban and rural population. Two of the municipalities will be divided into two clusters, and four smaller municipalities were combined to two clusters to account for the unequal population size. Services within hospitals and municipalities are free of charge in Denmark, and the responsibility for rehabilitation is shared between sectors [12]. At the time of discharge, older adults with a medically assessed need for rehabilitation are offered a referral for municipal rehabilitation [12]. A list of study sites can be obtained on request from the corresponding author.

### Eligibility criteria

The trial will include older adults 65 years of age and older, admitted to the ortho-geriatric ward with a hip fracture and residing in one of the municipalities. Other inclusion criteria are patients able to speak and understand Danish and orientated in time and place. Patients discharged to permanent residence in nursing homes or patients with competing diseases disabling relevant conversation, such as progressed dementia, or receiving palliative care, will be excluded.

### Who will take informed consent

Assessment of eligibility and informed consent was obtained up to 72 h post-surgery, by trial data collectors. In cases where cognitive function was medically unresolved, decisions on inclusion were done in discussions with nurses and physiotherapists at the ward and patients’ next of kin. Prior to obtaining written consent, patients will receive written and oral information as required by the regional ethics committee. The consent form developed by the national ethics committee in Denmark was used.

### Additional consent provisions for collection and use of participant data and biological specimens

Data will be collected in accordance with the Danish Data Protection Agency (20-21854). As required by Danish legislation, written informed consent will be obtained from participants to permit the collection of information from medical records.

### Intervention

#### Usual rehabilitation and care in the primary and secondary sectors

After admission to the emergency department, patients with a hip fracture are transferred to an ortho-geriatric ward. During hospitalisation, the patients are examined by an orthopaedic surgeon and a geriatric specialist. Mobilisation and rehabilitation are initiated within 24 h post-surgery and performed along with vital signs measurement for the early detection of critical illness and complication, throughout the entire hospitalisation period. A physiotherapist is responsible for rehabilitation which comprises walking, exercise, and instruction to a self-training programme. The Cumulated Ambulation Score (CAS) [13] is assessed daily and the need for walking aids is continuously evaluated. The rationale for usual praxis during admission is that early mobilisation and exercise, as well as early detection of critical illness, provide an optimal basis for regaining walking ability and reducing mortality. In the primary sector, usual rehabilitation varies in both content and setting. Content can vary in frequency of rehabilitation sessions and in focus of the session (e.g. gait, walking on stairs, and sit-to-stand at home) [9]. Rehabilitation is typically twice a week, completed in the patients’ own home or at a rehabilitation centre and with a duration of 6–8 weeks [9]. Rehabilitation can be supplemented with restorative care aimed to maintain activities of daily living (ADL). In both rehabilitation and restorative care, the older adults’ motivation is obtained by exercising specific ADL tasks. Care in the primary sector follows the plan prescribed by the hospital, and treatment changes have to be prescribed by the general practitioner.

#### Intervention description

The intervention will be offered to the intervention group in addition to the usual rehabilitation and care. The intervention is comprehensive and includes rehabilitation, empowerment, and care. The duration of the intervention will be 12 weeks (post-surgery). The basis for the intervention is that continuous and progressive rehabilitation, as well as early detection of critical illness and complication during and after hospitalisation, will improve the older adults’ physical performance. The older adults are expected to be motivated by an empowerment-orientated praxis [14].

Within the first 2 weeks after discharge, rehabilitation in the primary sector comprises five rehabilitation sessions. This will be followed by supervised rehabilitation twice a week for another 10 weeks. The rehabilitation in both sectors will follow a progressive rehabilitation programme including resistance exercise. Progression of resistance follows the national guideline for hip fractures which suggests resistance is added at 3 sets of 15 unweighted repetitions and progresses to 3 sets of 8 repetitions maximum [7]. For patients with a CAS ≥ 4, rehabilitation in a municipal rehabilitation centre will be recommended; alternatively, the resistance exercises will be performed at home with wrist weights. Except for the sit-to-stand exercise, the older adults will be requested to perform the exercises as often as possible, preferably three times a week. The exercise sit-to-stand as many times as possible will be recommended after each of the three main meals a day [15].

In the municipalities, nurses will visit the older adults on the third day after discharge to measure vital signs. Vital signs consist of early detection of illness or complications and pain management, e.g. blood pressure, pulse, respiratory frequency, saturation, consciousness, temperature, and saturation combined with measurement of C-reactive protein and haemoglobin.

An empowerment-orientated practice requires a change in the healthcare professionals’ approach towards seeing the older adults as a partner capable of acting and taking responsibility [16]. To implement the empowerment-orientated praxis, two initiatives are used: first, the patients will be given access to knowledge [16]. The older adults will receive a trolley containing the rehabilitation regime, exercise equipment, and a guide, targeted patient and next-of-kin to a digital healthcare app. The app contains videos and informative interviews with doctors and nurses from the ortho-geriatric ward and health professionals from the municipalities [17, 18]. Second, health professionals will participate in a workshop where they will learn about empowerment and how to use it. During the workshop, the health professionals will also be informed on the importance of strength training and measuring vital signs and pain and introduced to the rehabilitation regime. The intervention is described using the Template for Intervention Description and Replication (TIDieR) [19] (Table 2).

**Table 2:** Description of intervention and comparator using TIDieR

	Rehabilitation for life	Usual rehabilitation and care
Why	Continuous and progressive rehabilitation as well as early detection of critical illness and complication during and after hospitalisation will improve the older adults’ physical performance and decrease mortality. Knowledge empowers older adults and facilitates a change in mindset among health professionals.	Activity-based rehabilitation restore and maintain the activities of daily living. Older adults’ need to regain functions creates motivation.
What	25 rehabilitation sessions with a physiotherapist over 12 weeks of these 5 within 2 weeks from discharge are planned. A virtual meeting between physiotherapist in the primary and secondary sectors and older adults is conducted in the 2 rehabilitation sessions after discharge. The suitcase contains knowledge and equipment the older adults need to take responsibility and perform daily exercises. Health professionals participate in a workshop. Early detection of critical illness and complications performed day 3 after discharge.	Older adults’ general amount of rehabilitation is approximately 1–2 rehabilitation sessions a week for 6–8 weeks. Care has to be prescribed.
Who provide	Physiotherapists, nurses, and social- and health assistants.	Physiotherapists, nurses, and social- and health assistants.
How	Face to face, virtual meetings, and app	Face to face.
Where	Ortho-geriatric ward, the patients’ home, and in the rehabilitation centres.	Ortho-geriatric ward, the patients’ home, and in the rehabilitation centres.
When and how much	*Weeks 1–2 after discharge:* *-* Five training sessions with a physiotherapist, duration up to 60 min. -One virtual meeting duration 30 min. -Vial measurements, duration up to 45 min. If necessary, one follow-up meeting with the municipal emergency nurse assessment, duration up to 45 min. Week 3 to week 12 after discharge: 2 weekly rehabilitation session with a duration up to 45 min is planned.	*During admission*: rehabilitation in the ortho-geriatric consist of a daily session with a physiotherapist duration of 30 min. *Week 1 after discharge:* -1 rehabilitation session duration up to 45 min. *Weeks 2–8 after discharge:* -1 or 2 weekly sessions of rehabilitation duration 45 min.
Tailoring	Patients with a CAS score ≥ 4 receive rehabilitation at a rehabilitation centre. Progression follows the national guidelines [7].	The patients rehabilitate at home or at a rehabilitation centre, pending on an individual assessment.

#### Criteria for discontinuing or modifying allocated interventions

Physiotherapists and nurses will be instructed to adapt the exercise to patience individual tolerance. This is to avoid unnecessary harm in terms of exercise-induced pain. Furthermore, patients and health personnel are taught to act and involve hospital doctors or general practitioners if medication needs to be modified.

#### Strategies to improve adherence to interventions

Adherence to interventions is monitored by the project group by telephone interview with patients every 2 weeks for the first 12 weeks after discharge. All patients will receive an exercise diary, and physiotherapists are required to fill in the progression in resistance weekly. Nurses are required to fill out a nursing diary.

#### Relevant concomitant care permitted or prohibited during the trial

No restriction on concomitant care was prohibited during the trial.

#### Provisions for post-trial care

No provisions or compensation will be paid by the trial.

### Outcome

#### Primary outcomes

The primary outcome for the physical function is Timed Up and Go [20] 8 weeks after discharge.

As the study is organised across two sectors, the CAS score measured 30 days after discharge makes a second primary outcome.

TUG is a valid and reliable test that measures the time it takes a person to get up from a chair with an armrest, walk 3 m, return to the chair, and sit [21]. The standard error of measurement (SEM) for patients with hip fractures is 11% [9]. It is hypothesised that patients in the intervention group will achieve a significantly reduced TUG score compared to usual care.

The CAS assesses mobility by (a) getting in and out of bed, (b) sit to stand, and (c) gait with a usual walking aid. It is hypothesised that a significantly larger number of patients in the intervention group will have a CAS = 6 at 30 days post-surgery compared to the control [22].

#### Secondary outcomes

*Physical function* will also be measured using the New Mobility Score (NMS, 0–9) and the 30-s sit-to-stand test (30s-CST). The NMS assesses the patients’ gait inside, outside, and during shopping [23], and the 30s-CST is a valid test that assesses lower body strength [24, 25].

*Activities of daily living* will be measured using Barthel-20 (0–20), which is a validated tool used to assess the patients’ need for help to perform activities of daily living [26].

#### Other outcomes

*Physical function* is measured using handgrip strength (HGS) which is a biomarker for ageing [27].

*Activities of daily living* will be measured using composite physical function (CPF, 0–24). CPF assesses the patients’ need for help to basic and instrumental activities of daily living [28].

*Pain* will be assessed using the 4-point Verbal Rating Scale (VRS, no pain, slight pain, moderate pain, and severe pain) [29].

*Readmission* will be measured 30 days after discharge.

*Mortality* will be assessed as an event 30 days after discharge and within the first year.

*Quality of life and pain* will be measured using the EuroQol Five-Dimension Questionnaire [30]. EQ-5D is a standardised questionnaire, used to assess the patients’ health-related quality of life and function [31].

*Empowerment* will be assessed using the patient activation measure (PAM) [32]. PAM includes thirteen questions addressing prevention and lifestyle changes.

*Fatigue* will be assessed using the Brief Fatigue Inventory (BFI) [33].

*Collaboration between health professionals* will be assessed using a questionnaire designed by Joint Action Analytics to measure the relational capacity [34] The questionnaires will be distributed before workshops and 3 months after the workshop.

*Costs information* will be collected for a cost-utility analysis [35]. Data from registries, municipalities, and hospitals are gathered retrospectively while information on carers’ and volunteers’ expenses in assisting the older adult in activities of daily living is gathered prospectively.

Costs information and information on the number of supervised training sessions, activity level, pain, place of rehabilitation, and the number of self-training sessions will be collected every 2 weeks for 12 weeks. The patients will be equipped with a diary as a memory aid.

### Participant timeline

A timeline and a description of the specific data collected at each time point are presented in Table 3.

**Table 3:** Forms and procedures adapted from the SPIRIT 2013 explanation and elaboration: guidance for protocols of clinical trials [11]

Time point			Post allocation							
Activity/assessment	Enrolment −***t***_**1**_	Allocation, 0	In-hospital, ***t***_**1**_	2 weeks, ***t***_**2**_	4 weeks, ***t***_**3**_	8 weeks, ***t***_**3**_	12 weeks, ***t***_**4**_	6 months, ***t***_**5**_	12 months, ***t***_**6**_	Event
Eligibility screen	X									
Informed consent	X									
Allocation		X								
Demography			X^a^							
TUG			X^a^			X	X	X		
CAS			X		X					
Barthel-20			X			X	X	X	X	
NMS			X			X	X	X	X	
HGS			X^a^			X	X	X	X	
30s-CST			X^a^			X	X	X	X	
EQ 5D			X			X	X	X	X	
CPF			X			X	X	X	X	
VRS			X	X	X	X	X	X	X	
PAM			X^a^			X	X	X	X	
BFI						X	X	X	X	
Care			X	X						
Co-morbidity			X							
Bioimpedance			X				X			
Operation			X							
Re-operation										X
Re-admission										X
Mortality										X

Care covers early detection of illness, complications, and pain management, e.g. blood pressure, pulse, respiratory frequency, saturation, consciences, temperature, and saturation

*TUG* Timed Up and Go, *CAS* Cumulated Ambulation Score, *Barthel-20* Barthel 20-Item Index, *NMS* New Mobility Score, *30s-CST* 30-s Chair Stand Test, *EQ 5D* EuroQol-5 domain, *CPF* composite physical function, *HGS* handgrip strength, *VRS* Verbal Rating Scale, *BFI* Brief Fatigue Inventory

^a^ Marked will be measured at discharge

### Sample size

The annual enrollment of patients with hip fractures from the six municipalities was a mean of 392. With an assumption that 50% of patients fulfilling the inclusion criteria (196 of 392), 48 patients will be available for inclusion every 3 months equal to eight patients per cluster. However, due to frailty, a 20% dropout is expected. Based on these assumptions, we expect approximately six patients per cluster every 3 months for the trial equal to a total of 330 patients.

The power calculation for the TUG is based on a reduction of 25% [36] and an estimated TUG score at discharge of 21.1 s (9.2) [37, 38]. With six patients per cluster every quarter, estimated power is 89%. Interclass coefficient [39] is 0.01, and *α* is 0.05. Thus, patient recruitment period will be 21 months.

For CAS, the power calculation is based on a 25% increase in the proportion of older adults who, 30 days post-surgery, have a CAS score = 6, power equals 90%.

### Recruitment

All older adults admitted to the ortho-geriatric ward will be assessed for inclusion consecutively by data collectors.

### Assignment of interventions: allocation

Randomisation will be done in advance using a balanced Internet-based randomisation list [40].

### Concealment method

Randomisation will be performed by opening a sequentially numbered opaque envelope every 3 months. A person with no patient contact and unfamiliar with the project will undertake this job.

### Implementation

After agreeing to participate, patients are assigned pending on home addresses. Patients’ home address will be concealed until informed consent was obtained and prefracture baseline data collected. The data collector will inform the patient of the assigned group.

### Assignment of interventions: blinding

#### Who will be blinded

Blinding is not possible as the health professionals need to know the older adults who are citizens in municipality randomised to intervention. Due to the visibility of intervention, it is not possible to blind the assessor either.

#### Unblinding

Not applicable.

### Data collection and management

#### Plans for assessment and collection of outcomes

Data collectors collect data in-hospital, and at 8 weeks, 12 weeks, and 6 months through home visits. Inter-rater reliability will be investigated. To promote data quality, assessors are trained and data collections forms and “how to” guides will be developed.

#### Plans to promote participant retention and complete follow-up

Only health professionals in the primary sector assigned for the workshop will have contact with the older adults assigned for intervention. At the time of the procedure, the project group will ensure that the collection of data at admission and 8 weeks later is not performed by the same project assistant, and the same applies for the following collection of data. In case of dropout, the reason for this will be examined.

#### Data management

To promote data quality and secure data, data collectors will use iPads and enter the data directly in secured servers. Every 3 months, the project manager perform completeness checks, and the entire project group is instructed to be aware of the data quality.

#### Confidentiality

The participants will be allocated an individual trial identification number, and the participant’s data will be stored on secured servers in accordance with national laws. The data will only be accessible to members of the project group.

#### Plans for collection, laboratory evaluation, and storage of biological specimens for genetic or molecular analysis in this trial/future use

This trial does not involve collecting biological specimens for storage.

## Statistical methods

### Statistical methods for primary and secondary outcomes

In the descriptive analyses, intervention, and controls will be described and compared to assess homogeneity. Categorical variables will be compared using the chi-square tests and Student’s *t*-test, or log-rank test will be used for continuous variables depending on the distribution (normal or not).

The effect of the intervention for continuous variables will be assessed using a linear mixed model with a random effect for each cluster and a fixed effect for each step of the stepped wedge model.

Categorical and ordinal data will be analysed using either a logistic or an ordinal logistic model. The experiences of the healthcare professional will be examined with a paired Student’s *t*-test.

### Interim analyses

No interim analysis has been planned because the interventions delivered have been proved feasible and safe for the intended population.

### Methods for additional analyses (e.g. subgroup analyses)

As an ancillary analysis, differences in effect pending on clusters will be examined.

### Methods in analysis to handle protocol non-adherence and any statistical methods to handle missing data

The analyses of outcomes follow the intention-to-treat principle. Missing outcomes will be imputed with multiple imputation [41]. For non-adherence to protocol, non-response analyses will be performed for excluded patients and non-completers. A per-protocol analysis will be conducted as a sensitivity analysis.

### Plans to give access to the full protocol, participant-level data, and statistical code

Anonymised data will be made accessible on reasonable request and in compliance with national laws.

### Oversight and monitoring

#### Composition of the coordinating centre and trial steering committee

The trial will be organised with a project group responsible for the day-to-day management, data collection, and deliveries of the trial. The project group plan to meet once a month. A steering committee consisting of stakeholders from hospital and municipalities provide oversight and meets quarterly with the project group. To secure the scientific quality, a research group consisting of a senior researcher will be established. The project group and research group plan meetings by demand but intend to meet at least two times a year. An implementation group consisting of physiotherapists and nurses from hospitals and municipalities will also be created. The implementation group will be the project group’s direct contact to the clinicians and offer a forum to overcome challenges and facilitate communication between sectors and municipalities. The implementation group and project group meet once every 2 months.

#### Composition of the data monitoring committee, its role, and reporting structure

A data monitoring committee was not deemed relevant as this is an implementation RCT. The interventions are feasible for the patient group and mainly consist of standardised exercise and enabling exercise by reducing the risk of medical complications and pain.

#### Harms

Given the feasibility of the intervention, no harms are expected.

#### Frequency and plans for auditing trial conduct

This will be done on a day to day basis and systematically every six month.

#### Plans for communicating important protocol amendments to relevant parties

Decision on important trial amendments has to be made by the steering committee and will be communicated to all relevant parties. The protocol in the clinical trials registry will be updated.

#### Dissemination plans

The results will be disseminated through peer-reviewed journals and other media.

## Discussion

The project aims to improve physical function in older patients after hip fracture. It is hypothesised that patients in the intervention group will gain a significantly improved physical function compared to patients following usual care.

In the trial, we want to empower patients to self-exercise and to continue exercising after the intervention has ended. We do not expect cognitively impaired patients will be empowered by the stimuli put forward and excluded patients with severe cognitive impairments.

Besides improved physical function, it is important to accentuate that the study operates across sectors and organisational conditions on which the design is based. A clear advantage of the cluster randomised stepped-wedge design is the implementation of the intervention at the end of the trial municipalities and hospitals. By randomising in clusters and introducing incremental rollout, issues such as impaired organisational commitment should be met [42]. Furthermore, the design has been used in previous trials working in the primary and secondary sectors [42, 43]. At the end of the project, the intervention is implemented offering a manual for how interventions may be implemented in other hospitals and municipalities [44]. A drawback of the design is the risk of unequal exposure to seasonal trends.

The primary time of interest was 8 weeks after discharge, because this is comparable to the average duration of usual rehabilitation in municipalities. Guidelines indicate that 50% of older adults after hip fracture have a need of a 12-week intervention in spite additional effect is unknown [7]. We therefore extended the intervention to 12 weeks to evaluate the additional effect.

The implementation of the intervention might pose some challenges due to the needed organisational changes. Furthermore, procedures to monitor the delivery of the intervention have been set up, in terms of structured telephone interviews every 2 weeks. We expect the content of the trolley in form of exercise diaries, information to apps, and exercise equipment will help empower patients and health professionals.

Data on older patients’ activity levels and function enable the evaluation of possible associations between functional improvement and an increase in the level of activity.

### Trial status

This is protocol version number 1 date 10 November 2020. Initiation of recruitment commenced on 01 October 2020, and the recruitment completion date will be 30 October 2022.

## Supplementary Information

**Additional file 1.** Checklist for protocol of a clinical trial.

## Data Availability

Data sharing does not apply to this article as no datasets were generated or analysed during the current study. Exercise regimes, consent forms, further descriptions, etc. can be acquired from the corresponding author.
